# Fluvoxamine and lycopene alleviate cisplatin-induced kidney fibrosis by modulating miR-21 and fibrotic signaling pathways

**DOI:** 10.1038/s41598-026-55689-1

**Published:** 2026-06-04

**Authors:** Sally E. Abu-Risha, Samia S. Sokar, Mai A. Mousa, Nagla A. El-Shitany, Alaa E. Elsisi

**Affiliations:** Department of Pharmacology & Toxicology, Faculty of Pharmacy, Tanta University, Tanta, 31527 Egypt

**Keywords:** Fluvoxamine, Lycopene, Cisplatin, Kidney fibrosis, Oxidative stress, miR-21, Molecular medicine, Nephrology, Kidney diseases

## Abstract

Cisplatin (Cis) is a widely utilized chemotherapy drug for managing various cancers, but its efficacy is limited by nephrotoxicity, which frequently results in kidney fibrosis. Oxidative stress, inflammation, and the fibrotic transforming growth factor beta/small mothers against decapentaplegic (TGF-β/SMAD) pathway are among the underlying mechanisms. Long-term exposure to Cis causes apoptosis, extracellular matrix deposition, and fibrosis. Fluvoxamine (FLV), a selective serotonin reuptake inhibitor and sigma‑1 receptor agonist, lycopene (LYC), a carotenoid antioxidant, and their combination showed a novel protective influence on kidney fibrosis in male albino rats induced by administering Cis (7 mg/kg) by intraperitoneal (IP) injection once weekly for 4 weeks. FLV (5 mg/kg), LYC (10 mg/kg), and their combination were administered orally (PO) once daily for four weeks. Treatment reduced blood urea nitrogen (BUN), serum creatinine (S.cr), nuclear factor kappa B (NF-κB), microRNA-21 (miR-21) levels, and the pro-apoptotic response reflected by decreased Bcl-2-associated X protein (Bax) immunoreactivity while enhancing antioxidant activity. They also attenuated fibrosis markers, including TGF-β1, SMAD3, collagen I (Col-I), and alpha-smooth muscle actin (α-SMA). FLV and LYC effectively attenuated Cis-induced kidney fibrosis through antioxidant, antiapoptotic, and antifibrotic mechanisms. These findings may provide a new therapeutic approach to counter Cis-induced nephrotoxicity in clinical settings.

## Introduction

Cisplatin is a potent antineoplastic agent utilized in treating various tumors, including testicular, lung, bladder, and gynecologic malignancies^1^. Regardless of the anticancer efficacy, its medical application is limited due to its notable side effects, especially nephrotoxicity^2^. Approximately 20–30% of patients undergoing Cis therapy exhibit an elevated level of serum creatinine and impaired urine concentrating capacity within 3–5 days of treatment^3^. Kidney failure or nephrotoxicity resulting from chemotherapy may restrict chemotherapy options^4^.

In addition, Cis can induce morphological and biochemical alterations in the renal tissues characterized by tubular damage, inflammation, and kidney fibrosis, a significant problem that increases chronic kidney disease (CKD) progression and, eventually, renal failure^5^. Inflammation and fibrosis are the two most important hallmarks of Cis-induced nephropathy^6^. Excess reactive oxygen species (ROS) generation can harm tubular cells and cause kidney injury in patients undergoing therapy with Cis^7^. Therefore, exploring strategies to mitigate the nephrotoxicity associated with Cis treatment is imperative.

Kidney fibrosis is a progressive condition resulting in kidney function deterioration and abnormal accumulation of extracellular matrix (ECM) components. CKD and renal fibrosis affect 50% of individuals above 70 years old and impact 10% of the global population^8^. Oxidative stress and activating profibrotic mechanistic pathways, including the TGF-β pathway, play a role in the pathogenesis of kidney fibrosis caused by Cis^9^. Therefore, exploring strategies to mitigate the nephrotoxicity associated with Cis treatment is imperative to preserve kidney function.

Fluvoxamine (FLV), a selective serotonin reuptake inhibitor (SSRI), has been utilized for four decades to treat obsessive-compulsive disorder, anxiety, and depression^10^. Fluvoxamine acts as a sigma-1 receptor agonist (S1R) that regulates adaptive and innate immunological responses^11^. These facts suggest the association between FLV and attenuated fibroblast activation and reduced fibrosis; as a result, S1R activation by FLV reduces cardiac fibroblast activation and fibrosis^12^. Fluvoxamine also alleviated bleomycin-induced lung fibrosis^13^. However, its role in the context of kidney fibrosis and its associated fibrotic pathways and microRNA regulation, such as miR-21, remains unexplored. So far, the effect of FLV on kidney fibrosis remains unknown. Also, S1R activation exerts antioxidant and antiapoptotic effects.

Lycopene (LYC), one of the many compounds known as carotenoids, is a lipid-soluble molecule found primarily in tomatoes. It exhibits antioxidant properties because of its minimal toxicity and strong ability to neutralize free radicals^14^ despite LYC’s properties that include antioxidant, anti-inflammatory, and cell growth-inhibiting effects^15–17^; various studies suggest that LYC has an antifibrotic impact as it alleviates hepatic fibrosis induced by carbon tetrachloride (CCl_4_), oral submucous fibrosis, and lung damage induced by bisphenol A^16,18,19^. Consequently, this study is intended to examine the ameliorative impact of LYC on reducing Cis-induced kidney fibrosis using a rat model. Moreover, its potential to interfere with fibrosis-associated molecular signals and microRNAs in kidney tissues has not been previously investigated.

Fluvoxamine was selected in the present study because, in addition to its established pharmacological use as a SSRI, it acts as a sigma‑1 receptor agonist and may modulate inflammation, oxidative stress, and apoptosis. Lycopene was selected as a natural carotenoid antioxidant with reported antifibrotic and free radical-scavenging properties. The rationale for combining both agents was based on their potentially complementary mechanisms in counteracting cisplatin-induced renal injury.

In particular, within the scope of this investigation, the aim is to assess the possible protective benefits of FLV, LYC, and their combination on Cis-induced chronic kidney fibrosis. Combining FLV and LYC is based on their distinct, complementary mechanisms, FLV targeting cellular stress via S1R activation and LYC offering a strong antioxidant defense. Evaluating both agents allows for a broader investigation of their potential protective roles.

This study investigates molecular mechanisms, such as oxidative stress, apoptotic markers, fibrotic signals, and miR-21 regulation, providing insights into novel therapeutic strategies against chemotherapy-induced kidney fibrosis.

## Materials and methods

### Animals

The study was approved by the Research Ethics Committee at the Faculty of Pharmacy, Tanta University, Egypt, which followed CIOMS guidelines (ethical code: TP / RE11/23 Ph-7). Fifty male albino rats, aged 8 to 10 weeks and with body weights ranging from 150 to 200 g, were sourced from the National Research Center in Cairo. The rats were housed in cages (*n* = 5 rats/cage) under controlled conditions, maintaining a humidity of 60 ± 10%, a temperature of 25 ± 2^°^C, and a 12-hour light-dark cycle. All the animals involved in the experiment were given unrestricted tap water and food availability. In order to prepare the animals for the experiment, they were allowed to acclimate for one week. During the acclimatization period, rats were maintained under the same controlled environmental conditions described above, handled daily, and monitored for general health before the start of the experiment. Adult male albino rats were used because this model is commonly employed in cisplatin-induced nephrotoxicity studies, provides reproducible renal injury, and helps reduce variability related to sex-associated hormonal fluctuations. Inclusion, exclusion, and randomization procedures were conducted in accordance with the ARRIVE guidelines 2.0. Male healthy animals within the selected weight range were included.

### Experimental design

Rats were randomly allocated into five groups of animals (*n* = 10/group), as shown in (Table 1), using a computer-generated randomization sequence prepared in Microsoft Excel with the RAND () function, to receive the following treatments for 4 weeks. The Cis (7 mg/kg, IP)^20^ once weekly for 4 consecutive weeks, while FLV (5 mg/kg, PO)^21^, and LYC (10 mg/kg, PO) were administered once daily^22^ for 4 weeks. The doses were selected according to previous studies. On Cis administration days, FLV and/or LYC were given approximately 2 h prior to Cis administration. The normal control group served as the negative control, whereas the Cis-treated group served as the positive disease control for cisplatin-induced kidney injury. The sample size was estimated using a power analysis conducted with GraphPad StatMate and kidney function parameters as the main continuous outcomes. Based on differences reported in previous cisplatin‑induced nephrotoxicity studies^23,24^, a medium‑to‑large standardized effect size (f ≈ 0.5) between groups, a two‑sided significance level of 0.05, and a statistical power of 0.80 were assumed that approximately 8–10 animals per group would be sufficient to detect such differences. On this basis, 10 rats per group were used to account for attrition, adhering to the principle of minimizing animal use.

**Table 1:** Experimental design.

Groups	Experimental design
Normal control	Rats were given 0.5% carboxymethylcellulose (CMC) PO daily for 4 weeks.
Cis	Rats were given Cis (7 mg/kg, IP) once weekly for 4 weeks.
FLV + Cis	Rats were given FLV (5 mg/kg, PO) once daily and Cis (7 mg/kg, IP) once weekly for 4 weeks. On Cis administration days, FLV was given 2 h prior to Cis administration.
LYC + Cis	Rats were given LYC (10 mg/kg, PO) daily and Cis (7 mg/kg, IP) once weekly for 4 weeks. On Cis administration days, LYC was given 2 h prior to Cis administration.
FLV + LYC+Cis	Rats were given FLV (5 mg/kg/day, PO), LYC (10 mg/kg/ day, PO), and Cis (7 mg/kg/week, IP) for 4 weeks. On Cis administration days, FLV and LYC were given approximately 2 h prior to Cis administration.

### Drugs and chemicals

Fluvoxamine was from Abbott Healthcare Products (BV, The Netherlands), Cis from Mylan SAS (Saint-Priest, France), and LYC was purchased from Changsha Staherb Natural Ingredients (≥ 90% purity, China).

### Sample collection and preparation

On the 29th day, at the end of the experiment, all rats were anesthetized using sodium secobarbital (50 mg/kg). Once the absence of reflex responses was confirmed, euthanasia was performed by cervical dislocation in accordance with institutional ethical guidelines. A cardiac puncture was performed to collect blood. Kidneys were taken for histological and biochemical analysis. Blood was collected using anticoagulant-free test vials. The samples were permitted to coagulate at 4^°^C for 15 min prior to centrifugation at 3000 r.p.m. utilizing a SiGmA 3K15 centrifuge for 15 min. Serum aliquots were preserved at -80^°^C for further investigation. The kidneys were meticulously separated from the connective tissue adhesion. Histological and immunohistochemical investigations were performed on the left kidney (*n* = 5/group), which was fixed in 10% neutral buffered formalin, while the right kidney was cryopreserved at -80 °C for further biochemical investigation^25^. The right kidney was homogenized in cold buffered phosphate (100 mM, pH 7, containing 2 mM EDTA) at 10 mL per gram of tissue for biochemical assays. The homogenate was stored at 4°C for 15 min after being centrifuged at 4000 r.p.m. The supernatant was collected for use as a biological marker. The Pierce™ BCA Protein Assay Kit (Thermo Scientific, Cat. No. 23227) was employed to quantify the total protein content in the supernatant, following the manufacturer’s guidelines. All biochemical data were expressed per mg protein and adjusted to total protein content, with bovine serum albumin (BSA) adjusted as the standard. Histopathological scoring, image analysis, and biochemical/molecular measurements were performed using coded samples without knowledge of group allocation.

### Assessment of BUN and S.cr levels

The concentrations BUN and S.cr were determined using spectrophotometry, employing biochemical kits from Biodiagnostic Co., Egypt (BUN kit, Catalogue no. UR 21 10; S.cr kit, Catalogue no. CR 12 51) following the manufacturer’s guidelines.

### Determination of kidney MDA and SOD contents

The kidney malondialdehyde (MDA) content and SOD activity were determined using kits from Biodiagnostic Co., Egypt (MDA kit, Catalogue no. MD 25 29; SOD kit, Catalogue no. SD 25 21). All assays followed the manufacturers’ instructions.

### Determination of TGF-β1 and SMAD3 levels

The manufacturer’s instructions were followed to measure the levels of TGF-β1 and SMAD3 in kidney homogenate utilizing a sandwich-ELISA kit obtained from Sunred Bio. Tech. Co., China. (TGF-β1, Catalogue no. DZE201110780; SMAD3 Catalogue no. DZE201115401).

### Assessment of kidney NF-κB and miR-21 genes using real-time quantitative (qRT-PCR)

The NF-κB and miR-21 gene expression levels were assessed via real-time (qPCR) analysis. The Mini RNeasy Kit (QIAGEN, Hilden, Germany) (Catalogue no. 74104) was utilized to isolate total RNA, including miRNA, from kidney samples. The kit of QuantiTect reverse transcription (QIAGEN, Hilden, Germany) (Catalogue no. EP0441) was utilized for cDNA synthesis from mRNA. Assessments of mRNA and miRNA were conducted utilizing primers acquired from Bio Basic, Inc., Canada. The amplification method employs a mixture of 15 µL, including 0.5 µL of each primer, 2 µL of cDNA, 7.5 µL of SYBR Green universal master mix (manufactured by Thermo Scientific, USA), and 4.5 µL of nuclease-free water. Melting curve assessment was conducted at 50–99 °C, with the initial phase consisting of a 10-second hold at 95 °C and a 15-second annealing period for 55 cycles at 60°C. Table 2 provides a list of primer sequences; particular primers were developed utilizing the software (Rotor-Gene Q Series 2.0.3), Build 2, to compute the ΔCt value. The number of cycles is indicated by the Ct value after the fluorescence curve achieves its baseline. The relative expression of mRNA and miRNA for ß-actin and U6, respectively, was measured for fold changes utilizing the 2^−ΔΔCt^ technique^26^.

**Table 2:** Primer sequences of specific genes.

Gene	Primer sequence (5’-3’)	Reference
Rat ß. actin	F: TCCTCCTGAGCGCAAGTACTCT	^59^
	R: GCTCAGTAACAGTCCGCCTAGAA	
NF-κB	F: GCAAACCTGGGAATACTTCATGTGACTAAG	^60^
	R: ATAGGCAAGGTCAGAATGCACCAGAAGTCC	
U6 (housekeeping)	F: GCTTCGGCAGCACATATACTAAAAT	^61^
	R: CGCTTCACGAATTTGCGTGTCAT	
miR-21	F: CGGCGGTAGCTTATCAGACTGATGT	^62^
	R: GTGCAGGGTCCGAGGT	

ß. actin, beta-actin; NF-κB, Nuclear factor-κB; miR-21, microRNA 21; F, forward; R, reverse.

### Histopathology examination

The formalin-fixed left kidney was divided longitudinally into two halves and paraffin-embedded. The kidney tissue segment (5 μm thick) was dyed differentially in two slide sets. The first batch of samples applied hematoxylin-eosin staining to be utilized for the light microscopic assessment of histological changes^27^. The assessment was carried out semi-quantitatively, placing scores from (0 to 4), with (0) representing normal kidney tissue, (1) indicating mild lesions, (2) signifies moderate lesions, (3) represents severe lesions, and (4) corresponds severe diffuse lesions. This scoring was based on various vascular alterations, including encompassing congestion and bleeding, hydronephrosis, degenerative lesions, and inflammatory alterations. Staining by Masson’s trichrome (MT) was employed to illustrate collagen fibers^25^. The positive expression area (%) was employed to evaluate the staining intensity of MT, and the software program Image J (NIH, USA) was used for image analysis and processing.

### Immunohistochemical examination

The immunohistochemical staining techniques were conducted following the guidelines established by Khalil et al.^28^. A 0.05 M citrate buffer at pH 6.8 was used to dewax the sections and immerse them in order to retrieve the antigens. Afterward, these sections were subjected to the protein-blocking solution containing 0.3% hydrogen peroxide. Subsequently, the tissue sections were treated with Bax monoclonal antibody (Invitrogen, MA5-14003, 1: 100), Col-I polyclonal antibody (Invitrogen, Cat #PA1-26204), α-SMA (Catalogue number MA5-11547, 1: 800 dilution), and E-cadherin monoclonal antibody (HECD-1) (Invitrogen, Cat # 13-1700). The samples were kept at room temperature for 30 min with a rat monoclonal secondary antibody (Cat# K3468, Dako EnVision+™ System with Horseradish Peroxidase Labelled-Polymer) and goat anti-rabbit secondary antibody (Cat# K4003, EnVision+™ System Horseradish Peroxidase Labelled Polymer; Dako) for polyclonal antibodies following rinsing with phosphate-buffered saline. A DAB kit was used to visualize the slides, and it was later stained with Mayer’s hematoxylin for contrast. The Bax antibody staining intensity was represented as a percentage of positive staining among 1000 kidney cells in 8 high-power fields. The analysis software Image J (NIH, USA) was employed to analyze the percent of positive expression per area (mm^2^) for Col-I, α-SMA, and E-cadherin immunostaining.

### Statistical analysis

GraphPad Prism 9 (San Diego, CA, USA) was used for data analysis, and sample size calculation was conducted using GraphPad StatMate v2 (San Diego, CA, USA). The Shapiro-Wilk test was applied to assess data normality. Tukey’s post-hoc test and the one-way ANOVA were used to examine parametric data. Results were presented as mean ± SD. The Kruskal-Wallis and Dunn’s tests were utilized for non-parametric statistical analysis of H&E histological lesions. The results were presented as median and range. Graphic representations were used to interpret the analyzed data visually. A P-value of less than 0.05 was considered statistically significant.

## Results

### Fluvoxamine, LYC, and their combination mitigated kidney injury and enhanced kidney functions

**Fig. 1:**
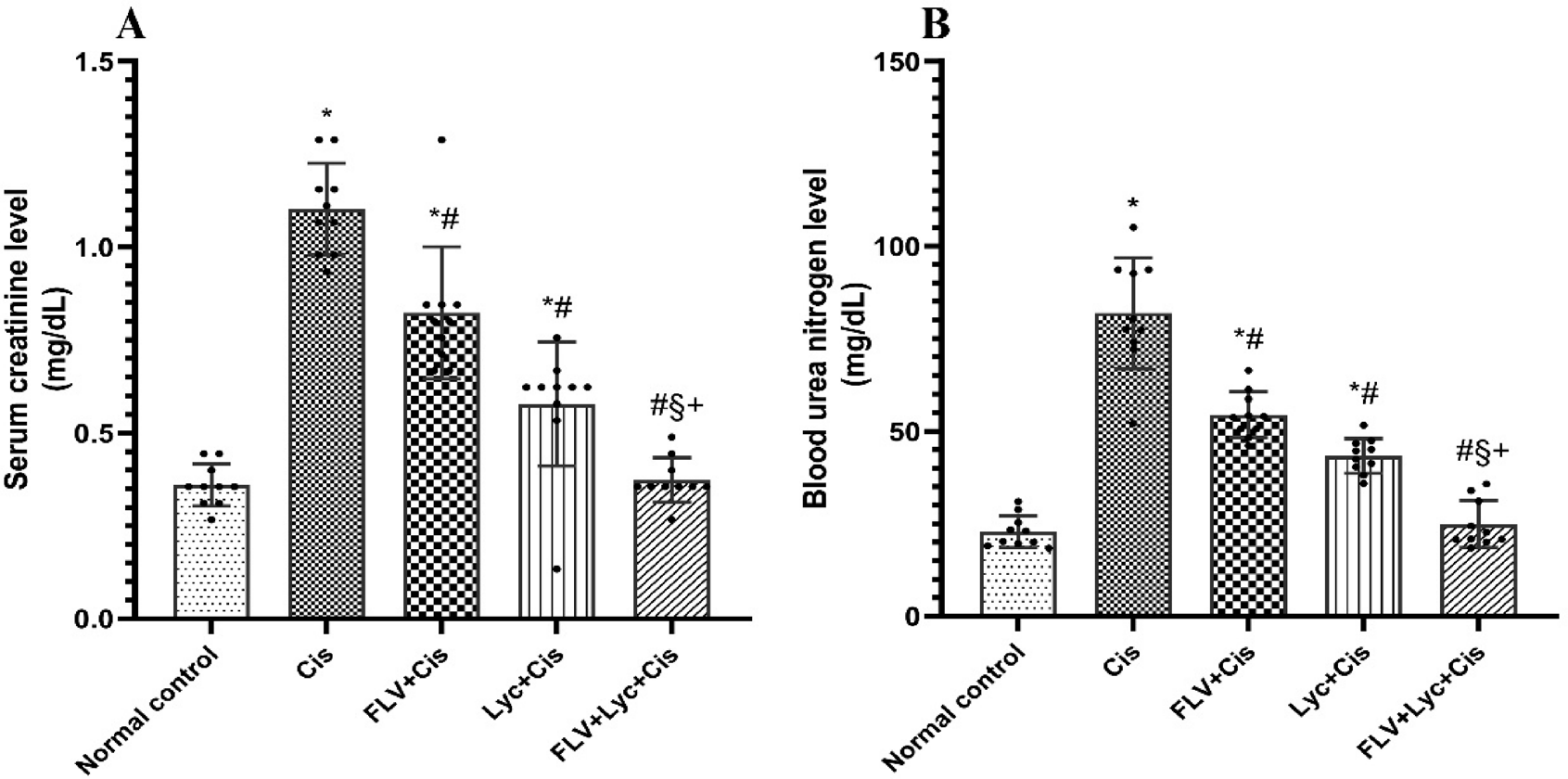
Effects of FLV, LYC, and their combination on serum BUN and creatinine levels in cisplatin-treated rats. (**A**) BUN level. (**B**) Serum creatinine. The data are expressed as mean ± SD. ^*^p < 0.05 compared to the normal control, ^#^*p* < 0.05 compared to the Cis group, ^§^*p* < 0.05 compared to the FLV + Cis, ^+^*p* < 0.05 compared to the LYC + Cis. Cis, cisplatin; FLV, fluvoxamine; LYC, lycopene.

To evaluate the extent of renal functional impairment induced by Cis and the possible protective effects of FLV and LYC, serum BUN and creatinine levels were assessed. BUN and S.cr levels in the Cis group increased significantly compared to the normal control group. Conversely, pretreatment with FLV, LYC, and FLV + LYC significantly reduced BUN and S.cr levels compared to the Cis group. Additionally, the FLV + LYC group significantly reduced kidney BUN and S.cr levels compared to either FLV or LYC groups (*p* < 0.05). The results indicated that FLV and LYC alone reduce BUN and serum creatinine levels. Also, the combination of FLV and LYC had the most pronounced effect, in which the kidney biomarkers were restored to their normal levels (Fig. 1A and B).

### Effects of FLV, LYC, and their combination on kidney oxidative stress biomarkers

**Fig. 2:**
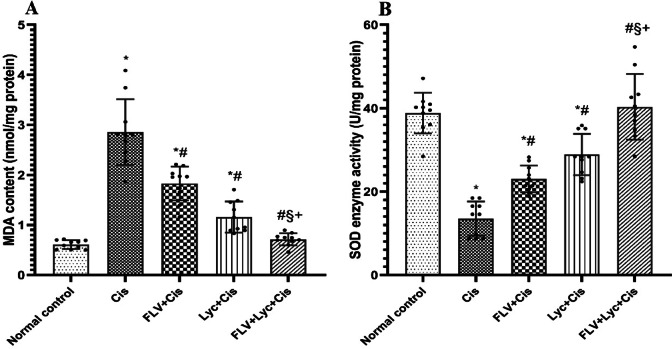
Effects of FLV, LYC, and their combination on MDA and SOD contents in cisplatin-treated rats. (**A**) MDA content. (**B**) SOD enzyme activity. The data are expressed as mean ± SD. ^*^p < 0.05 compared to the normal control, ^#^*p* < 0.05 compared to the Cis group, ^§^*p* < 0.05 compared to the FLV + Cis, ^+^*p* < 0.05 compared to the LYC + Cis. Cis, cisplatin; FLV, fluvoxamine; LYC, lycopene.

To investigate the contribution of oxidative stress to Cis-induced renal injury and the potential antioxidant effects of the tested treatments, kidney MDA content and SOD activity were measured. MDA and SOD biomarkers were measured to examine the oxidative stress. The MDA contents significantly increased in the Cis group, as shown in (Fig. 2A). Additionally, FLV, LYC, and FLV + LYC significantly decreased the MDA contents in comparison with the Cis group. Conversely, SOD in the Cis group, activity was notably reduced relative to the normal control group. However, pretreatment with FLV, LYC, and FLV + LYC led to a significant rise in SOD activity when compared to the Cis group (Fig. 2B). Additionally, the FLV + LYC group significantly reduced MDA content compared to either FLV or LYC groups, and increased SOD activity compared to either FLV or LYC groups.

### Effect of FLV and LYC on TGF-β1 and SMAD3 contents

**Fig. 3:**
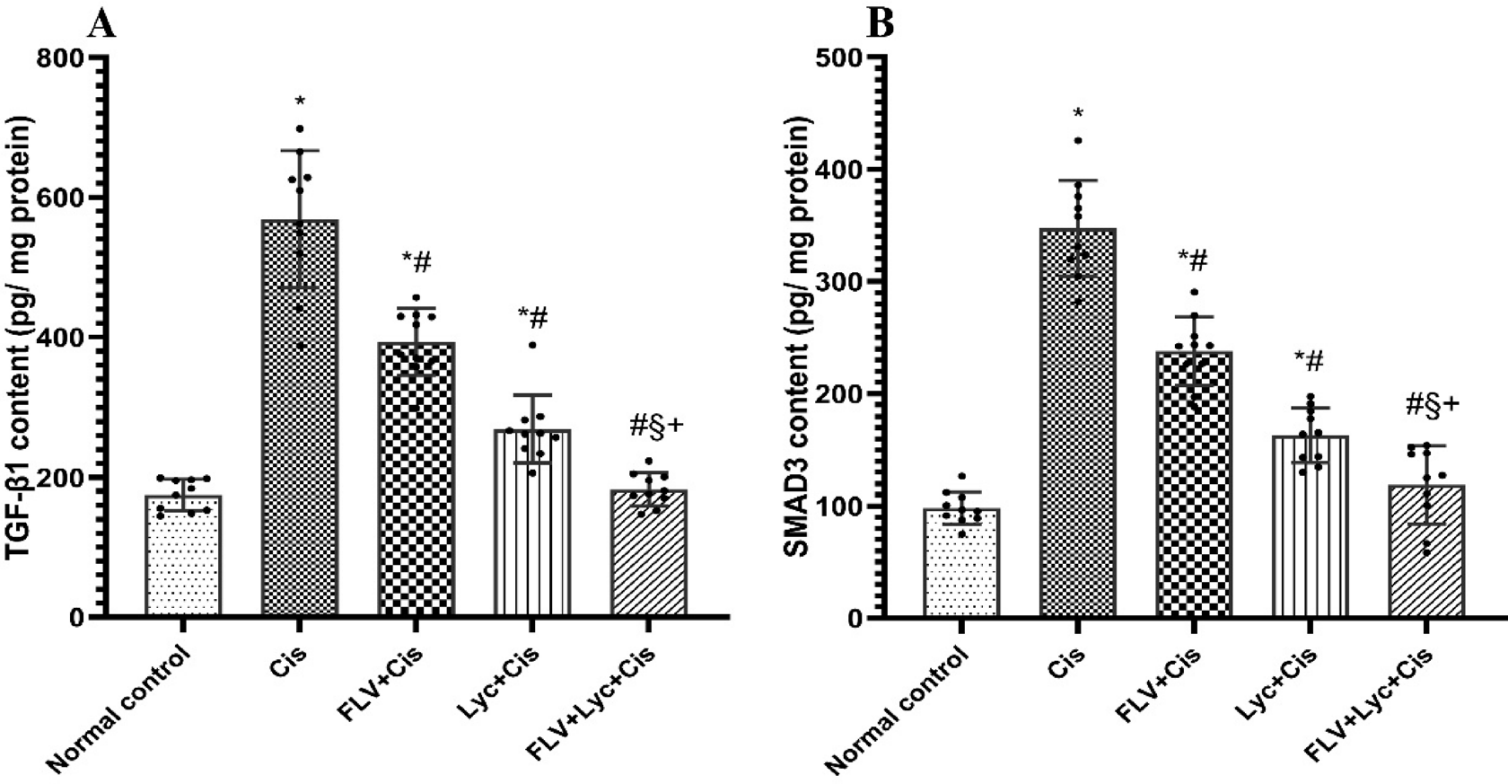
Effects of FLV, LYC, and their combination on TGF-β1 and SMAD3 levels in cisplatin-treated rats. (**A**) TGF-β1 level. (**B**) SMAD3 level. The data are expressed as mean ± SD. ^*^p < 0.05 compared to the normal control, ^#^*p* < 0.05 compared to the Cis group, ^§^*p* < 0.05 compared to the FLV + Cis, ^+^*p* < 0.05 compared to the LYC + Cis. Cis, cisplatin; FLV, fluvoxamine; LYC, lycopene.

To assess the involvement of profibrotic signaling in Cis-induced kidney fibrosis, TGF‑β1 and SMAD3 levels were determined. As presented in (Fig. 3), TGF-β1 and SMAD3 contents in the Cis group increased significantly relative to the normal control group. However, administrating FLV, LYC, and FLV + LYC resulted in significant reductions in TGF-β1 and SMAD3 contents relative to the Cis group (*p* < 0.05) (Fig. 3A and B). Conversely, the FLV + LYC group significantly reduced kidney TGF-β1 and SMAD3 contents relative to either FLV or LYC groups. The results indicate that FLV and LYC alone reduce TGF-β1 and SMAD3 contents. Also, the combination of FLV and LYC had the most pronounced effect, resulting in a more significant reduction of TGF-β1 and SMAD3 contents than either treatment alone.

### Effects of FLV, LYC, and their combination on NF-κB and miR-21 mRNA expression levels

**Fig. 4:**
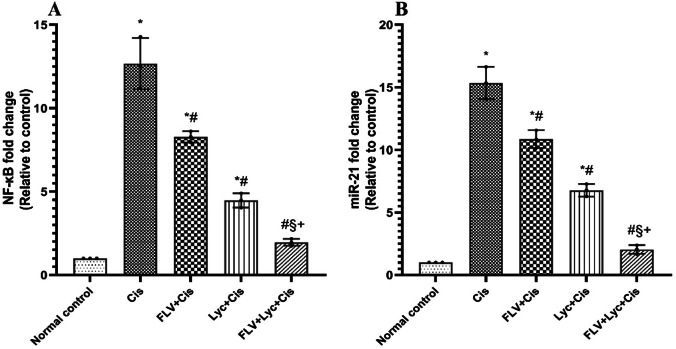
Effects of FLV, LYC, and their combination on mRNA gene relative expression of NF-κB and miR-21 in cisplatin-treated rats. (**A**) NF-κB fold change. (**B**) miR-21fold change. The data are expressed as mean ± SD. ^*^p < 0.05 compared to the normal control, ^#^*p* < 0.05 compared to the Cis group, ^§^*p* < 0.05 compared to the FLV + Cis, ^+^*p* < 0.05 compared to the LYC + Cis. Cis, cisplatin; FLV, fluvoxamine; LYC, lycopene.

To explore the molecular mechanisms associated with inflammation- and fibrosis-related gene regulation, the expression levels of NF‑κB and miR‑21 were evaluated. Furthermore, the Cis-intoxicated group induced significant increases in kidney NF-κB (Fig. 4A) and miR-21 (Fig. 4B) gene expressions when compared to the normal control group. However, Pretreatment with FLV, LYC, and FLV + LYC significantly decreased the NF-κB and miR-21 gene expression relative to the Cis group (Fig. 4A and B). Additionally, the FLV + LYC group significantly reduced kidney NF-κB and miR-21 expression compared to using either FLV or LYC.

### Effects of FLV, LYC, and their combination on kidney histopathological changes

**Fig. 5:**
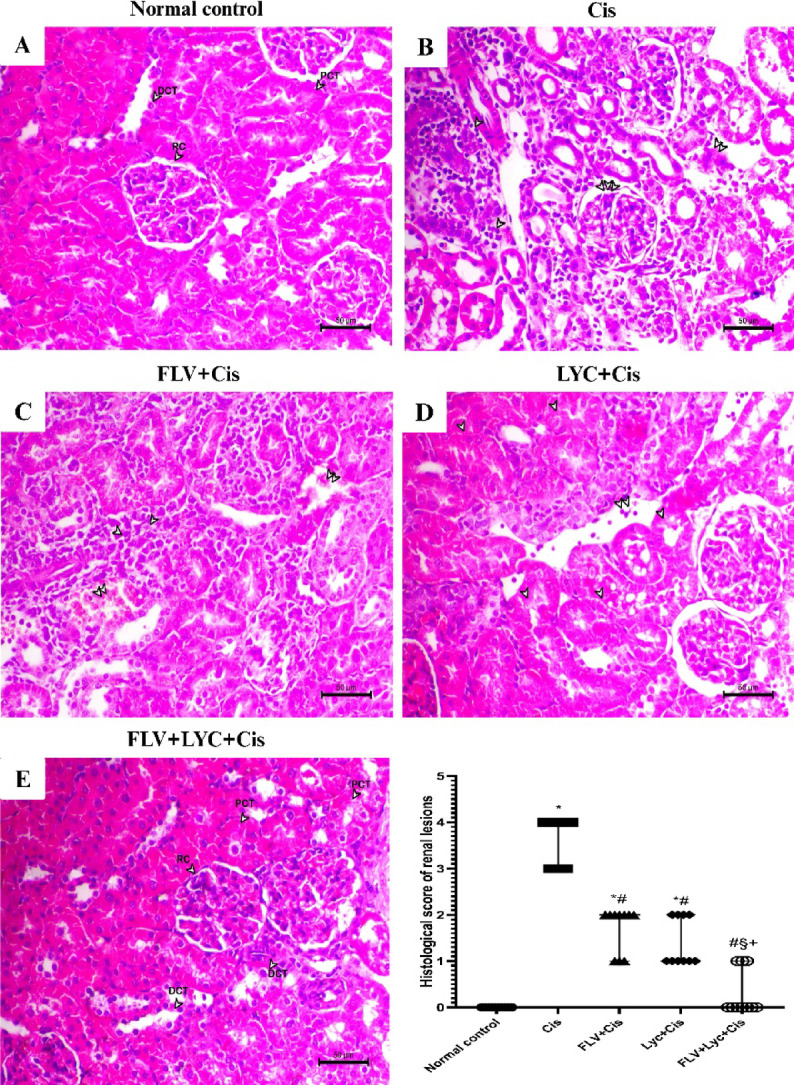
Kidney sections histopathological microscopic analysis (H&E, x200, bar = 50 μm). (**A**) The normal control group shows a well-preserved kidney architecture, including intact renal corpuscle (RC), distal convoluted tubules (DCT), and proximal convoluted tubules (PCT) (arrowheads). (**B**) The Cis group exhibits interstitial nephritis, leukocyte infiltration (double arrowheads), degeneration, and necrosis of tubular epithelial cells (double arrowheads). (**C**) The FLV + Cis group shows reduced interstitial nephritis, leukocyte infiltration (arrowheads), and tubular epithelial cell degeneration and necrosis (double arrowheads). (**D**) The LYC + Cis group demonstrates a decrease in interstitial nephritis, healthier kidney tubular epithelium, and reduced necrosis (arrowheads), with minimal leukocyte infiltration (double arrowheads). (**E**) The FLV + LYC+Cis group exhibits an absence of interstitial nephritis, an increase in healthy tubular epithelial cells within the proximal (PCT), distal convoluted tubules (DCT), and minimal necrosis and degeneration (arrowheads), along with normal renal corpuscle structure. (**F**) Semi-quantitative scoring of histopathological lesions; presented as median and range. ^*^p < 0.05 compared to the normal control, ^#^*p* < 0.05 compared to the Cis group, ^§^*p* < 0.05 compared to the FLV + Cis, ^+^*p* < 0.05 compared to the LYC + Cis. Cis, cisplatin; FLV, fluvoxamine; LYC, lycopene.

To examine structural renal alterations induced by Cis and the effect of treatment on tissue architecture, histopathological changes were evaluated in hematoxylin and eosin (H&E)-stained kidney sections. Histopathological evaluation in the control group revealed normal kidney architecture (Fig. 5A). The Cis group revealed marked interstitial nephritis, leukocyte infiltration, degeneration, and necrosis of kidney tubular epithelial cells (Fig. 5B,F). Unlike the Cis group, FLV significantly reduced interstitial nephritis, leukocyte infiltration, and kidney tubular epithelial cell degeneration and necrosis (Fig. 5C,F). The interstitial nephritis was significantly reduced in the LYC + Cis group, with an increase in healthy kidney tubular epithelial cells and a reduction in necrosis and degeneration. Also, leukocyte infiltration is minimal (Fig. 5D,F). The FLV + LYC + Cis group showed an absence of interstitial nephritis, a significant increase in healthy kidney proximal convoluted tubules (PCT) and distal convoluted tubules (DCT) (Fig. 5E), along with a significant decrease in necrosis and degeneration, aside from the typical configuration of kidney corpuscles, unlike the Cis group (Fig. 5F).

### Effects of FLV, LYC, and their combination on collagen area (%)

**Fig. 6:**
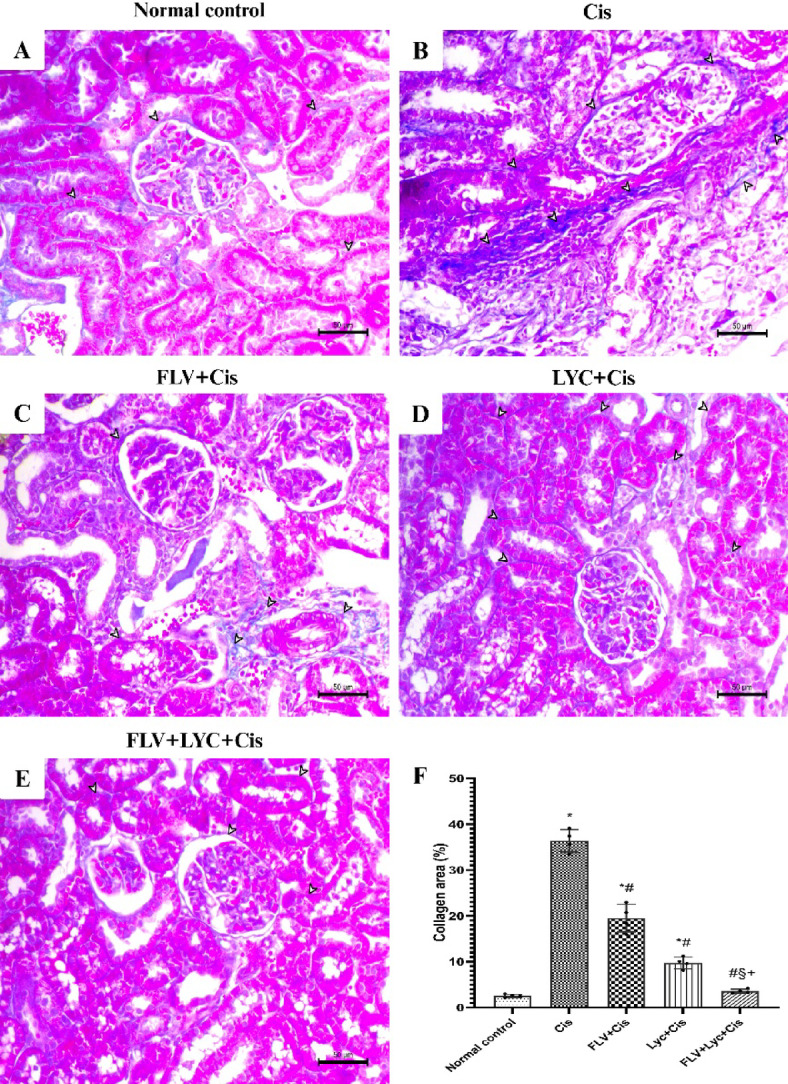
Masson’s trichrome stain of kidney sections (_X_200, bar = 50 μm). (**A**) The normal control group exhibits minimal fibrotic tissue. (**B**) The Cis group indicates an abundance of fibrous tissues around and between kidney tubules. Indicated by blue staining (arrowheads). (**C**) The FLV + Cis group showed a decrease in fibrous tissues around the kidney glomeruli and within the perivascular area (arrowheads). (**D**) The LYC + Cis group shows mild periglomerular and peritubular fibrosis (arrowheads). (**E**) The FLV + LYC+Cis group shows a marked decrease in periglomerular and interstitial fibrous tissues (arrowheads). (**F**) all data are presented as mean ± SD. ^*^p < 0.05 vs. normal control, ^#^*p* < 0.05 vs. Cis, ^§^*p* < 0.05 vs. FLV + Cis, ^+^*p* < 0.05 vs. LYC + Cis. Cis, cisplatin; FLV, fluvoxamine; LYC, lycopene.

To further assess collagen deposition and the degree of fibrosis, kidney sections were stained with Masson’s trichrome (MT). Kidney sections stained with MT showed the cortex from the control group rats had a few numbers of fibrous tissues. MT was used to stain the collagen fibers with blue (Fig. 6A). The Cis group showed an abundance of fibrous tissues around and between kidney tubules (Fig. 6B). The FLV + Cis group exhibited a decrease in fibrous tissues around the kidney glomeruli and within the perivascular area (Fig. 6C). The LYC + Cis group showed mild periglomerular and peritubular fibrosis (Fig. 6D). The FLV + LYC + Cis group exhibited a marked decrease in periglomerular and interstitial fibrous tissues (Fig. 6E).

Post hoc analysis demonstrated that the Cis group exhibited a significantly higher level of MT staining than the normal control group. However, pretreatment with FLV, LYC, and FLV + LYC significantly decreased MT staining in comparison with the Cis group. In contrast, the FLV + LYC-treated group notably reduced kidney MT staining compared to using either FLV or LYC groups (Fig. 6F).

### Fluvoxamine, LYC, and their combination suppressed kidney apoptosis in Cis-intoxicated rats

**Fig. 7:**
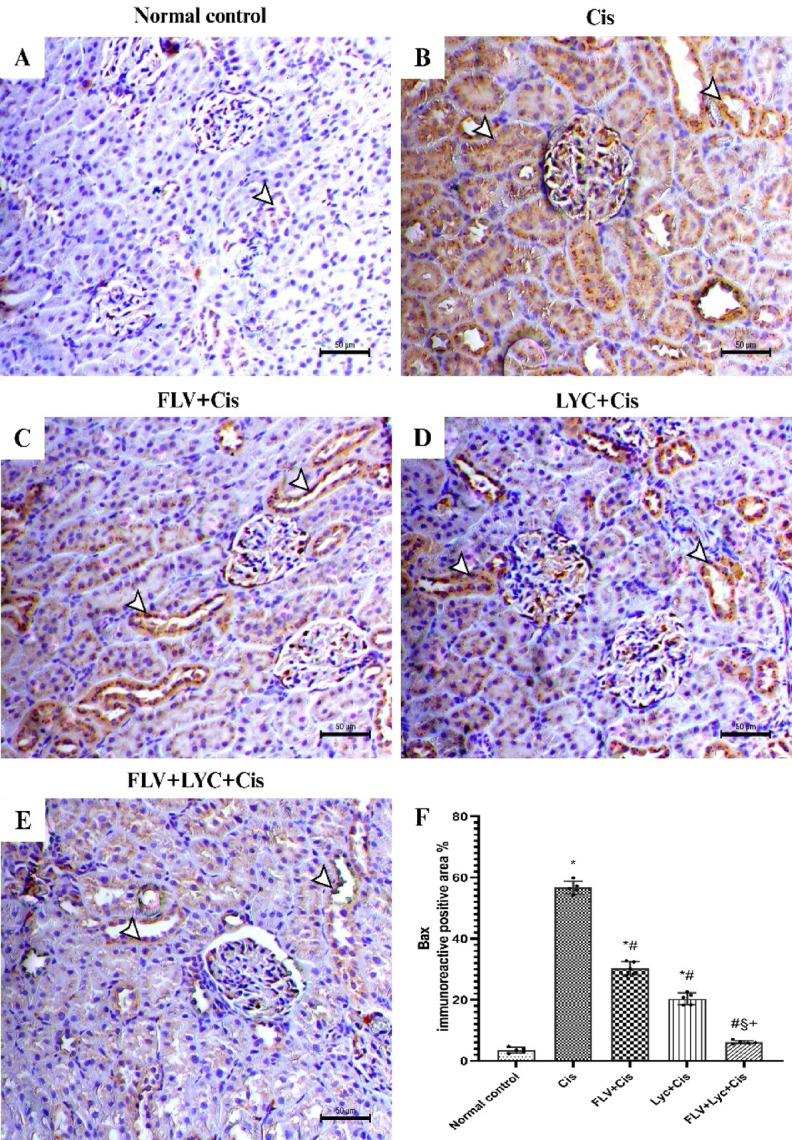
Immunohistochemical staining of Bax of kidney sections (_X_200, bar = 50 μm). (**A**) The normal control group showed mild expression of Bax in the tubular epithelium (arrowhead). (**B**) The Cis group demonstrates a significant rise in the Bax immunostaining inside the tubular epithelium (arrowheads). (**C**) The FLV + Cis group exhibits reduced expression of Bax within the tubular epithelium (arrowheads). (**D**) The LYC + Cis group demonstrates a reduction in the expression of Bax immunostaining within the tubular epithelium (arrowheads). (**E**) The FLV + LYC+Cis group shows a minimal Bax expression (arrowheads); (**F**) All data are given as mean ± SD. ^*^p < 0.05 vs. normal control, ^#^*p* < 0.05 vs. Cis, ^§^*p* < 0.05 vs. FLV + Cis, ^+^*p* < 0.05 vs. LYC + Cis. Cis, cisplatin; FLV, fluvoxamine; LYC, lycopene.

Bax immunostaining was evaluated in kidney tissue sections to determine whether Cis-induced kidney injury was associated with enhanced tubular apoptosis and whether FLV and LYC could mitigate this response. Immunohistochemical examination of kidney sections from normal control animals showed mild stains of Bax within the kidney tubular epithelium (Fig. 7A). On the other hand, kidney sections from rats given Cis exhibited a significant rise in kidney tubular epithelium Bax immunostaining (Fig. 7B). Kidney sections from rats pretreated with FLV showed decreased expression of Bax within the kidney tubular epithelium (Fig. 7C). Bax expression within the kidney tubular epithelium was found to be reduced in the LYC + Cis group (Fig. 7D). The kidney of the FLV + LYC+Cis group exhibited a significant reduction in the Bax staining in the kidney tubular epithelium (Fig. 7E).

Regarding the Bax immunoreactive area (mm^2^), Cis-treated rats showed a significantly higher area relative to the normal control group; however, the FLV, LYC, and FLV + LYC significantly decreased Bax staining compared to the Cis group (*p* < 0.05). On the other hand, the FLV + LYC group significantly reduced kidney Bax staining compared to using either FLV or LYC groups (Fig. 7F).

### Effects of FLV, LYC, and their combination on Col-I in kidney tissue

**Fig. 8:**
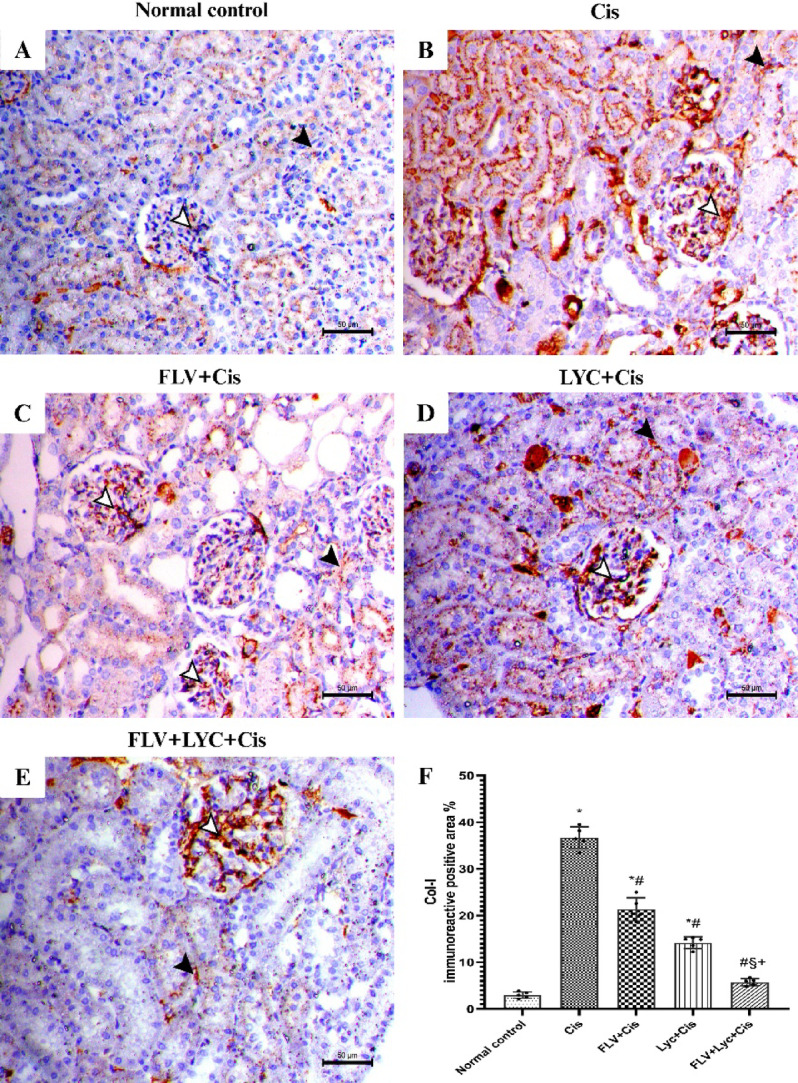
Immunohistochemical staining of Col- I of kidney sections (_X_200, bar = 50 μm). (**A**) The normal control group exhibits a mild glomerular and peritubular expression of Col- I antibody within the kidney parenchyma (white and black arrowheads). (**B**) The Cis group demonstrates increased glomerular and interstitial Col- I immunostaining (white and black arrowheads). (**C**) The FLV + Cis shows a reduction in the expression of Col- I in kidney parenchyma (white and black arrowheads). (**D**) The LYC + Cis group presents a significant reduction in Col- I antibody immunostaining expression (white and black arrowheads). (**E**) The FLV + LYC+Cis group shows a minimal expression of the Col- I antibody (white and black arrowheads); (**F**) All data are given as mean ± SD. ^*^p < 0.05 vs. normal control, ^#^*p* < 0.05 vs. Cis, ^§^*p* < 0.05 vs. FLV + Cis, ^+^*p* < 0.05 vs. LYC + Cis. Cis, cisplatin; FLV, fluvoxamine; LYC, lycopene.

Excessive deposition of Col‑I is a hallmark of renal fibrosis, Col‑I immunostaining was examined to assess the impact of Cis and the potential antifibrotic effects of FLV and LYC on collagen accumulation in the kidney. Kidney sections immunostained with the Col-I antibody of the control animal showed mild glomerular and peritubular expression within the kidney parenchyma (Fig. 8A). The Cis group showed marked glomerular and interstitial Col-I immunostaining within the kidney tissues (Fig. 8B). The kidney of FLV + Cis exhibited a reduction in Col-I expression among the kidney parenchyma (Fig. 8C). The kidney of the LYC + Cis group showed decreased expression of Col-I within the kidney parenchyma (Fig. 8D). The kidneys of the FLV + LYC+Cis group exhibited a significant reduction in Col-I immunostaining inside the kidney parenchyma (Fig. 8E).

The Col- I immunoreactive area (mm^2^) of Cis-treated rats showed a significantly higher area relative to the normal control group. However, pretreatment with FLV, LYC, and FLV + LYC notably decreased Col- I staining compared to the Cis group; in contrast, using FLV + LYC significantly reduced Col-I staining compared to FLV or LYC groups (Fig. 8F).

### Effects of FLV, LYC, and their combination on α-SMA in kidney tissue

**Fig. 9:**
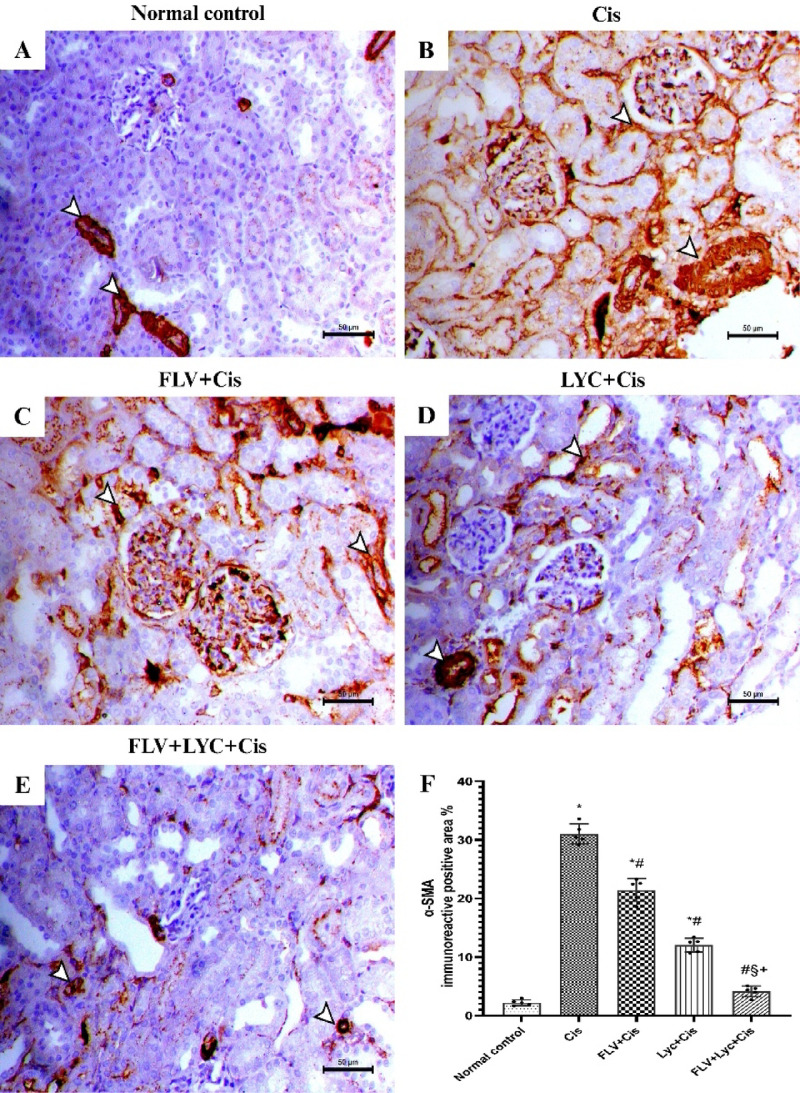
Immunohistochemical staining of α-SMA of kidney sections (X200, bar = 50 μm). (**A**) The normal control group exhibits a mild expression of α-SMA within the kidney parenchyma, localized only to vascular smooth muscle (arrowheads). (**B**) The Cis group exhibits a marked increase in the periglomerular, vascular, and interstitial α-SMA immunostaining (arrowheads). (**C**) The FLV + Cis group demonstrates a reduction in the expression of α-SMA in the kidney parenchyma (arrowheads). (**D**) The LYC + Cis group shows a reduction in the expression of α-SMA (arrowheads). (**E**) The FLV + LYC+Cis group presents a minimal α-SMA antibody immunostaining, primarily restricted to the blood vessel walls (arrowheads); (**F**) All data are given as mean ± SD. ^*^p < 0.05 vs. normal control, ^#^*p* < 0.05 vs. Cis, ^§^*p* < 0.05 vs. FLV + Cis, ^+^*p* < 0.05 vs. LYC + Cis. Cis, cisplatin; FLV, fluvoxamine; LYC, lycopene.

Expression α‑SMA was assessed to evaluate the degree of myofibroblast activation and interstitial fibrosis after Cis treatment and its modulation by FLV and LYC. Sections of the normal control group exhibited mild expression of α-SMA among the kidney parenchyma. The positive expression was noticed only within the medial layer of the kidney blood vessels (Fig. 9A). In the Cis group, α-SMA immunostaining showed a notable rise in the periglomerular, vascular, and interstitial regions of the kidney tissues (Fig. 9B). The kidney of FLV + Cis exhibited a reduction in the immunostaining of the α-SMA among the kidney parenchyma (Fig. 9C). A decrease in α-SMA staining was observed in the kidney parenchyma of the LYC + Cis group (Fig. 9D). The kidney of the FLV + LYC+Cis group revealed a pronounced decline in α-SMA staining in the kidney parenchyma (Fig. 9E).

As illustrated in (Fig. 9F), the α-SMA immunoreactive area (mm^2^) of Cis-treated rats showed a significantly higher area relative to the normal control group. In contrast, administration of FLV, LYC, and FLV + LYC significantly decreased α-SMA staining in comparison to the Cis group (*p* < 0.05). In contrast, FLV + LYC significantly reduced α-SMA staining compared to either FLV or LYC groups.

### Effects of FLV, LYC, and their combination on E-cadherin in kidney tissue

**Fig. 10:**
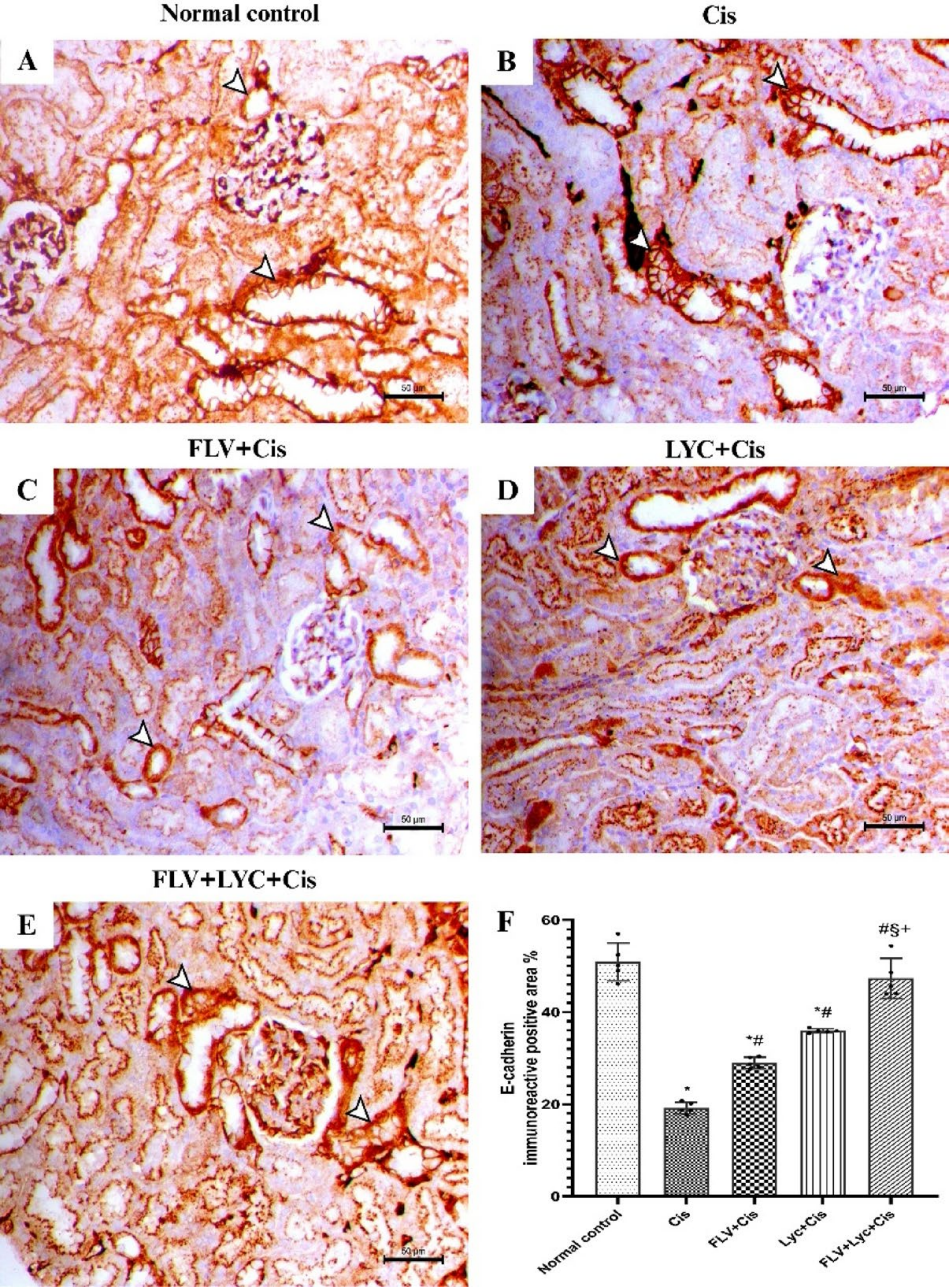
Immunohistochemical staining of E-cadherin stain in kidney sections (X200, bar = 50 μm). (**A**) The normal control group exhibits prominent peritubular and interstitial expression of E-cadherin within the kidney parenchyma. (**B**) The Cis group exhibits a substantial reduction in E-cadherin immunostaining. (**C**) The FLV + Cis group demonstrates increased expression of E-cadherin within the kidney parenchyma. (**D**) The LYC + Cis group exhibits an increased expression of E-cadherin. (**E**) The FLV + LYC+Cis group shows a marked restoration in E-cadherin expression within the kidney parenchyma. (**F**) All data are given as mean ± SD. ^*^p < 0.05 vs. normal control, ^#^*p* < 0.05 vs. Cis, ^§^*p* < 0.05 vs. FLV + Cis, ^+^*p* < 0.05 vs. LYC + Cis. Cis, cisplatin; FLV, fluvoxamine; LYC, lycopene.

E‑cadherin immunostaining was evaluated to investigate whether Cis-induced fibrosis involved disruption of epithelial integrity and whether FLV and LYC could preserve tubular epithelial phenotype. kidney sections from control animals (Fig. 10A) showed marked peritubular and interstitial expression of E-cadherin within the kidney parenchyma. The Cis group showed a notable reduction in E-cadherin immunostaining (Fig. 10B). The kidney of FLV + Cis showed increased E-cadherin expression within the kidney parenchyma (Fig. 10C). The kidney of the LYC + Cis group exhibited a considerable rise in the E-cadherin staining within the kidney parenchyma (Fig. 10D). The kidney of the FLV + LYC+Cis group resulted in a significant increase in E-cadherin staining among the kidney parenchyma (Fig. 10E).

Additionally, Cis-treated rats showed a significantly lower area % of E-cadherin relative to the normal control group; however, prior administration of FLV, LYC, and FLV + LYC significantly increased E-cadherin staining relative to the Cis group (*p* < 0.05). FLV + LYC markedly elevated E-cadherin staining compared to using FLV or LYC groups. The results indicated that both FLV and LYC alone increased E-cadherin. Also, a combination of FLV and LYC had the most pronounced effect, resulting in a more significant increase than either treatment alone (Fig. 10F).

## Discussion

**Fig. 11:**
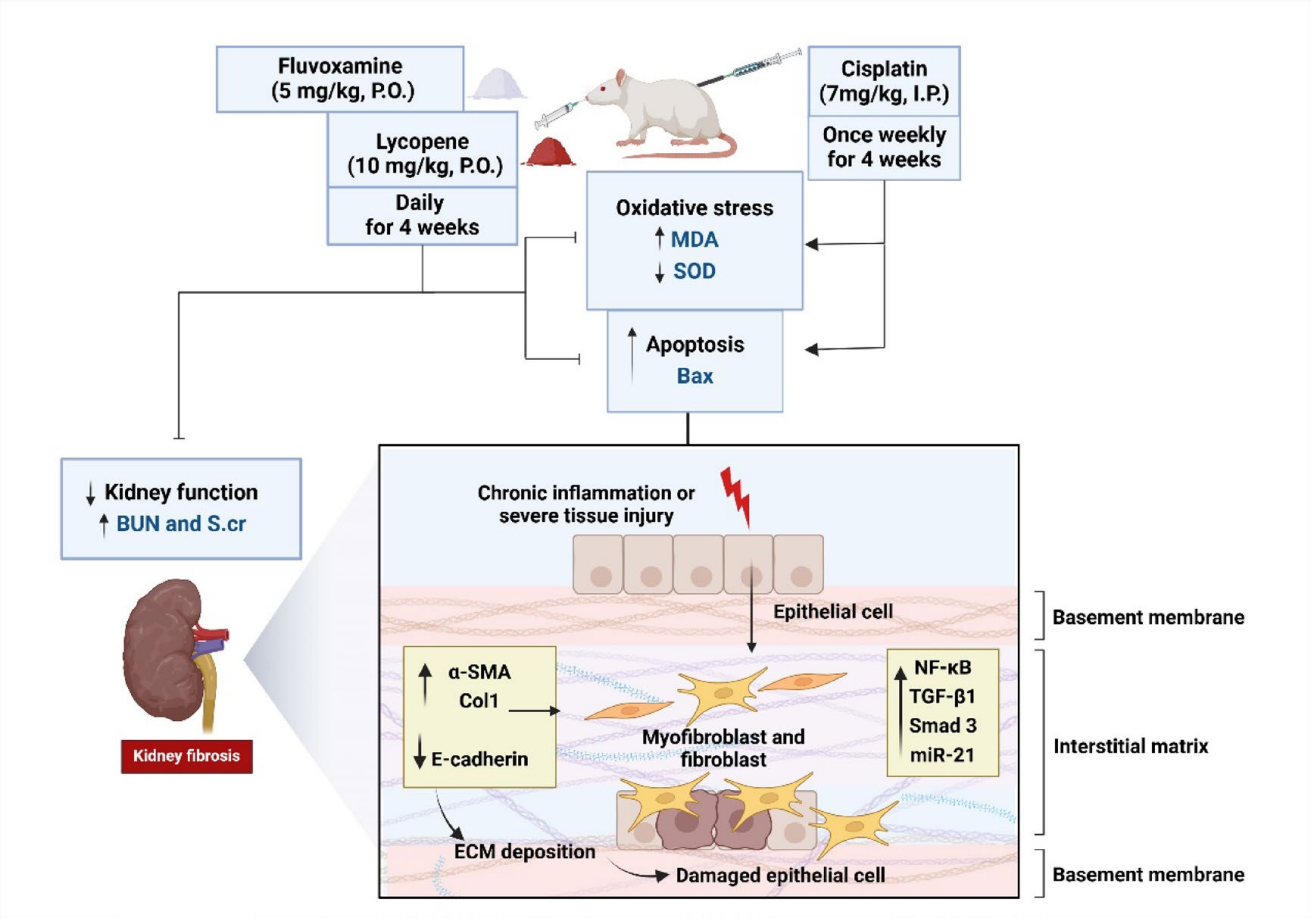
Kidney fibrosis triggered by cisplatin and the preventive roles of fluvoxamine and lycopene. MDA, malondialdehyde; SOD, superoxide dismutase; Bax, Bcl-2-associated X protein; TGF-β1, transforming growth factor- β1; miR-21, microRNA-21; SMAD3, Mothers against decapentaplegic homolog 3; Col- I, collagen- I; α-SMA, α- smooth muscle actin; NF-κB, nuclear factor-κB.

This study demonstrated the protective effect of FLV, LYC, and their combination against Cis-induced kidney fibrosis. The treatment reduced S.cr and BUN levels, suppressed oxidative stress markers, pro-apoptotic Bax immunoreactivity and reversed fibrosis-related changes, including the expression of TGF-β1, SMAD3, Col-I, α-SMA, and MT staining, while enhancing E-cadherin expression. FLV and LYC modulated molecular pathways, including NF-κB and miR-21 expression, and enhanced antioxidant activity. These results underline the effectiveness of FLV and LYC in mitigating kidney fibrosis through antioxidant, antiapoptotic, and antifibrotic effects (Fig. 11).

Kidney fibrosis is a key characteristic of CKD, and it represents the standard terminal pathway toward progressive deterioration in renal function. This process is defined by the accumulation of ECM proteins, mainly collagen deposition, in renal interstitial and glomerular compartments. This scarring process significantly diminishes the normal kidney architecture and function of filtering blood. The progression of kidney fibrosis can ultimately progress to end-stage renal disease (ESRD), characterized by significant loss of function, which results in dialysis or transplantation, often linked to a high rate of death and illness^29^.

Cisplatin is a highly active chemotherapeutic agent applied in the management of diverse cancer types, including ovarian, lung, and testicular cancer. Its efficacy is derived from its capacity to disrupt the DNA of cancer cells, thereby preventing their replication and resulting in the reduction or elimination of the tumor.

The use of Cis is restricted by its nephrotoxicity despite its anticancer properties^30^. It triggers oxidative stress along with inflammation in the kidneys, which can result in chronic kidney injury and fibrosis. Over time, this fibrosis can impede kidney function, potentially leading to CKD^31^. In addition, Cis treatment can frequently create psychological stress or depression as a result of its adverse effects and the emotional toll of cancer therapy^32^. Identifying the underlying mechanisms involved in developing nephroprotective treatments was the primary objective of mitigating these adverse effects. This study was meant to explore the protective effect of FLV, LYC, and their combination.

The current study examined the molecular pathways through which these drugs work as antifibrotics. Also, it demonstrated that Cis induced kidney fibrosis and declined kidney performance through a significant increase in S.cr and BUN levels. These findings agree with prior studies^33,34^. Pre-administration of FLV, LYC, and their combination attenuated fibrosis and enhanced overall kidney function by mitigating the rise in S.cr and BUN.

Fluvoxamine activates S1R and modulates inflammatory activity in cell and animal inflammation models^35^. Using a low dose of FLV as an antidepressant can help regulate mood and reduce anxiety while limiting potential medication interactions or adverse consequences^32^. Studies have demonstrated that FLV possesses both antioxidant and anti-inflammatory effects^36^. Using FLV can safeguard the kidneys from the damaging impact of Cis, which is principally caused by the formation of ROS and inflammatory reactions.

Lycopene is a potent antioxidant derived from tomatoes. Studies have demonstrated its ability to defend cells from oxidative harm induced by ROS^37^. Also, recent studies show that LYC may have antidepressant properties. It reduces depression caused by persistent stress^38^. Therefore, the pretreatment of both drugs, either alone or in combination, may mitigate kidney injury.

Reactive oxygen species are essential in developing kidney fibrosis because they induce oxidative stress, which activates profibrotic pathways and causes cellular injury^39^. Excessive ROS generation results in lipid peroxidation, DNA harm, and protein modifications, leading to cellular death and tissue injury. This injury initiates inflammatory responses, and the persistent production of ROS results in the release of signaling molecules and growth regulators, including TGF-β^40^. Recent research findings indicate that decreasing the formation of ROS might play a key role in managing and avoiding kidney fibrosis linked to Cis administration^41^.

The formation of ROS generated by Cis modifies the Kelch-like ECH-associated protein (Keap)/nuclear factor erythroid 2-related factor 2 (Nrf2)/heme oxygenase-1 (HO-1) pathway that correlates with a decline in overall antioxidant potential^42^. Our results indicated a marked rise in MDA contents and a decrease in SOD function relative to the control rats. In contrast, pretreatment with FLV, LYC, and their combination significantly decreased MDA contents and enhanced SOD activity. Prior research has demonstrated that FLV and LYC have oxidative stress-reducing characteristics^43^.

**Fig. 12:**
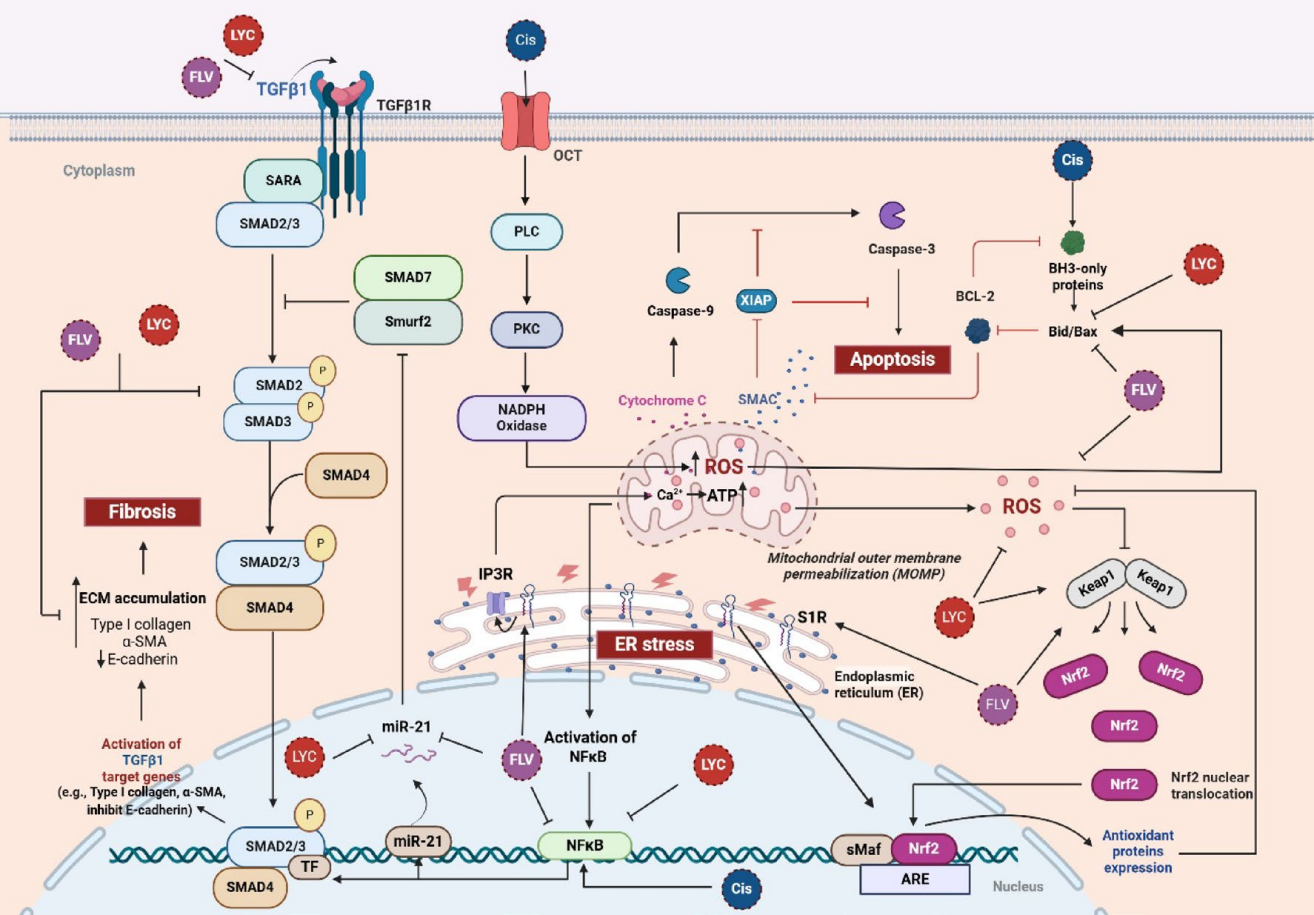
The Protective effects of fluvoxamine and lycopene through different mechanisms in counteracting cisplatin-induced kidney fibrosis. Cisplatin activates PLC and PKC in kidney cells via OCTS, leading to NADPH oxidase activation. ROS production results in ER stress, which decreases mitochondrial membrane permeability and releases cytochrome C and pro-apoptotic factors. Oxidative stress disrupts Keap1-Nrf2 interaction. Cisplatin also stimulates NF-κB, which increases miR-21 level that causes downregulation of SMAD7 and promotes fibrosis through the TGF-β/SMAD3 pathway. Fluvoxamine exerts a renoprotective effect by stimulating ER S1R, balancing calcium release via IP3R, and preventing cytoplasm and mitochondrial calcium overload-inducing oxidative stress. It also activates Nrf2 and stimulates nucleus-based SOD and antioxidant genes enzymes, and downregulates TGF-β/SMAD3 and miR-21 to mitigate fibrosis. Lycopene complements these effects by activating Nrf2, counteracting oxidative damage, suppressing NF-κB and miR-21, and downregulating the TGF-β/SMAD3 pathway. Fluvoxamine and lycopene combination prevent fibrosis by reversing the profibrotic markers. Cis, cisplatin; FLV, fluvoxamine; LYC, lycopene; PLC, phospholipase C; PKC, protein kinase C; OCT, organic cation transporters; NADPH, Nicotinamide adenine dinucleotide phosphate; ROS, reactive oxygen species; ER, endoplasmic reticulum; Cyto C, cytochrome c; Bax, Bcl-2-associated X protein; Nrf2, nuclear factor erythroid 2-related factor-2; Keap1, Kelch-like ECH-associated protein 1; SOD, superoxide dismutase; NF-κB, nuclear factor-kappaB; miR-21, microRNA-21; TGF-β1, transforming growth factor- β1; SMAD, Mothers against decapentaplegic homolog 3; ECM, extracellular matrix; α-SMA, α- smooth muscle actin; Col- I, collagen- I; S1R, sigma-1 receptor; IP3R, Inositol trisphosphate receptor.

The novelty of this work lies in the combined action of FLV and LYC in preventing kidney fibrosis induced by Cis. Although the previous research has primarily focused on the effects of antioxidants or anti-inflammatory agents individually, our findings demonstrate that the combined application of these agents reduces fibrosis markers and affects microRNA expression, particularly miR-21, which has not been previously correlated with Cis-induced fibrosis. This approach provides a more comprehensive strategy for preventing kidney fibrosis in this model, in addition to regulating the TGF-β/SMAD pathway. The summarized mechanistic processes are illustrated in (Fig. 12).

In addition, Cis-induced cell death, produced by apoptosis of tubular cells in the kidney, leads to renal tubular atrophy^44^. Apoptosis occurs through the discharge of mitochondrial cytochrome c via Bax, oxidative stress, and inflammatory cytokines. Bcl-2 prevents the triggering of the caspase-3 pathway and the mitochondrial cytochrome c release^45^. In Cis-intoxicated rats, FLV, LYC, and their combination reduced Bax in the kidney, demonstrating a strong antiapoptotic effect. In accordance with the current findings, the FLV-mediated activation of Sig-1R reduces neuronal cell death due to ER stress^46^. Furthermore, LYC elevated Bcl-2 protein levels, reduced Bax, and activated cleaved caspase-3 in the kidney tissue of mice^47^.

In addition to oxidative stress, microRNAs (miRNAs) play a crucial role in the development of kidney fibrosis. The study of microRNAs (miRNAs) has seen an unparalleled surge in research in the past several years, driven by the pressing necessity to identify the genes they target and the diseases to which they are relevant. Research in the field of miRNAs has recently uncovered crucial roles for some miRNAs in cellular processes, including differentiation, proliferation, immune responses, and development, as well as in the control of genes connected to human illnesses^48^. Recent results indicate that important microRNAs are extensively produced within renal tissue and influence the impact of TGF-β1 activity in CKD. This is particularly relevant to renal researchers. So, to uncover the process underlying fibrogenesis, it would be a significant and promising endeavor to examine microRNA expression patterns in the Cis-induce kidney fibrosis model^49^.

One of these miRNAs is miR-21; the current results indicated a significant upregulation in NF-κB and miR-21 expression in the Cis group. Oxidative stress plays an essential impact in the progression of kidney fibrosis by regulating miRNA expression, particularly miR-21. Our study found that Cis-induced oxidative stress upregulates miR-21 expression, activating profibrotic pathways. This finding aligns with Amini et al.^50^, which demonstrated the renoprotective effects of naringin and trimetazidine on renal ischemia/reperfusion damage in rats via inhibiting apoptosis and downregulating micoRNA-10a. Interestingly, FLV and LYC reduced miR-21 expression in Cis-treated rats, suggesting that antioxidants may mitigate fibrosis by targeting both oxidative stress and miR-21 regulation. Modulation of miR-21 through antioxidant treatment offers a promising therapeutic approach for kidney fibrosis. Other research has also revealed overexpression of miR-21 in the kidneys of mice with Cis nephrotoxicity; suppression of miR-21 significantly lowered EMT gene upregulation produced by Cis^51^. These results suggest that miR-21 functions as a key mediator in Cis nephrotoxicity and a possible target for novel therapies to alleviate kidney fibrosis caused by Cis. Activation of NF-κB resulted in increased miR-21 expression and suppression of SMAD7 (a negative regulator of SMAD3), thereby initiating profibrotic signaling pathways through TGF-β expression and the phosphorylation of SMAD3, which associates with SMAD4 to modulate the transcription of profibrotic TGFβ1 target genes related to ECM elements such as α-SMA, Col- I, and E-cadherin^52^. Our research demonstrated that FLV, LYC, and their combination attenuated NF-κB and miR-21 expression, and this finding has not been previously documented in the literature.

The TGF-β/SMAD pathway, along with α-SMA, Col-I, and E-cadherin in kidney tissue, were evaluated to investigate the potential antifibrotic effects of the studied drugs. Kidney fibrosis is characterized by the overproduction of TGF-β and the excessive, sustained accumulation of ECM components^53^. Accordingly, our results indicated that Cis-induced kidney fibrosis exhibited a significant increase in TGF-β1 and SMAD3 levels and overexpression of Col- I, α-SMA, while decreased E-cadherin expression than the untreated group, and these findings, in accordance with other research^54^. In contrast, administrating FLV, LYC, and their combination significantly downregulated TGF-β1, SMAD3, Col- I, and α-SMA while increasing E-cadherin expression. Our findings align with prior reports of the antifibrotic effects of FLV and LYC in the lung^55^, cardiac^56^, oral submucous^57^, and liver fibrosis^58^. Additionally, tissue analysis confirmed the antifibrotic effects of FLV, LYC, and their combination. The data indicate that FLV and LYC’s antioxidant, antiapoptotic, and antifibrotic characteristics may contribute to their potential protective effects against Cis-induced kidney fibrosis. This study has some limitations. First, FLV and LYC were evaluated only as pretreatments, and we did not investigate whether administration after the onset of cisplatin-induced injury would provide similar protection; therefore, their efficacy in established nephrotoxicity remains uncertain. Second, we used a single dose level and schedule for both agents and did not perform a formal dose–response analysis. In particular, FLV was administered at a relatively low dose (5 mg/kg) in order to minimize potential adverse effects or drug–drug interactions, but further studies are needed to determine the full safety profile, optimal dosing, and timing of FLV and LYC in different experimental settings. These issues should be considered in future investigations to better define the translational potential of FLV and LYC in Cis-induced kidney injury.

## Conclusion

This study identified the potential fibrotic pathway involved in kidney fibrosis induced by Cis administration. In contrast, pretreatment with FLV, LYC, and their combination mitigated kidney impairments, improved oxidative stress, apoptosis, fibrosis, and reduced kidney injury in the groups induced by Cis. Furthermore, FLV, LYC, and their combination diminished the TGF-β1/SMAD3 pathway. The combination of FLV and LYC had the most substantial impact, leading to a significant impact compared to each therapy alone. The main mechanisms responsible for the beneficial effects of FLV and LYC include the inhibition of miR-21 production, modulation of the TGF-β1/SMAD3 pathway, reduction of ROS, and attenuation of apoptotic signaling.

## Data Availability

Data generated during and/or analyzed in this study can be obtained from the corresponding author upon reasonable request.
